# Clinical features and risk factors for post-partum depression in a large cohort of Chinese women with recurrent major depressive disorder

**DOI:** 10.1016/j.jad.2011.06.047

**Published:** 2012-02

**Authors:** Tian Tian, Yihan Li, Dong Xie, Yifeng Shen, Jianer Ren, Wenyuan Wu, Chengbin Guan, Zhen Zhang, Danning Zhang, Chengge Gao, Xiaoming Zhang, Jinbo Wu, Hong Deng, Gang Wang, Yunshu Zhang, Yun Shao, Han Rong, Zhaoyu Gan, Yan Sun, Bin Hu, Jiyang Pan, Yi Li, Shufan Sun, Libo Song, Xuesheng Fan, Yi Li, Xiaochuan Zhao, Bin Yang, Luxian Lv, Yunchun Chen, Xiaoli Wang, Yuping Ning, Shenxun Shi, Yiping Chen, Kenneth S. Kendler, Jonathan Flint, Hongjun Tian

**Affiliations:** a Tianjin Anding Hospital, No. 13 Liu Lin Road, Hexi District, Tianjin 300222, PR China; b Wellcome Trust Centre for Human Genetics, Oxford OX3 7BN, UK; c Fudan University Affiliated Huashan Hospital, No. 12 Wulumuqi Zhong Road, Shanghai 200040, PR China; d Shanghai Jiao Tong University School of Medicine affiliated Shanghai Mental Health Centre, No. 600 Wan Ping Nan Road, Shanghai 200030, PR China; e Shanghai Tongji University affiliated Tongji Hospital, No. 389 Xinchun Road, Shanghai 200065, PR China; f Nanjing Brain Hospital, No. 264 Guangzhou Road, Nanjing, Jiangsu 210029, PR China; g No. 4 Affiliated Hospital of Jiangsu University, No. 246 Nan Men Da Street, Zhenjiang, Jiangsu 212001, PR China; h Shandong Mental Health Center, No. 49 East Wenhua Road, Jinan, Shandong 250014, PR China; i No. 1 Hospital of Medical College of Xian Jiaotong University, No. 277 West Yan Ta Road, Xi'an, Shaanxi 710061, PR China; j No. 1 Hospital of Zhengzhou University, No. 1 East Jianshe Road, Zhengzhou, Henan 450052, PR China; k No. 1 Mental Health Center Affiliated Harbin Medical University, No. 23 You Zheng Jie, Nangang District, Harbin, Heilongjiang, PR China; l Mental Health Center of West China Hospital of Sichuan University, No. 28 Dian Xin Nan Jie, Wu Hou District, Chengdu, Sichuan 610041, PR China; m Beijing Anding Hospital, Capital Medical University, No. 5 An Kang Hutong Deshengmen wai, Xicheng District, Beijing 100088, PR China; n Hebei Mental Health Center, No. 572 Dongfeng Road, Baoding, Hebei 071000, PR China; o Shengjing Hospital of China Medical University, No. 36 Sanhao Street, Heping District Shenyang, Liaoning 110817, PR China; p Shenzhen Kangning Hospital, No. 1080, Cui Zu Street, Luo Hu, Shenzhen 518020, PR China; q No. 3 Affiliated Hospital of Sun Yat-sen University, No. 600 Tian He Road, Tian He District, Guangzhou, Guangdong 510630, PR China; r No. 1 Hospital of Shanxi Medical University, No. 85 Jiefang South Road, Taiyuan, Shanxi 030001, PR China; s Mental Hospital of Jiangxi Province, No. 43 Shangfang Road, Nanchang, Jiangxi 330029, PR China; t The First Affiliated Hospital of Jinan University, No. 613 West Huangpu Avenue, Guangzhou 510630, PR China; u Wuhan Mental Health Center, No. 70, You Yi Road, Wuhan 430022, PR China; v No. 3 Hospital of Heilongjiang Province, No. 135 Jiao Tong Lu, Beian, Heilongjiang, PR China; w Jilin Brain Hospital, No. 98 Zhong Yang Xi Lu, Siping, Jilin 136000, PR China; x The First Hospital of China Medical University, No. 155 Nanjing Bei Jie, He Ping District, Shenyang 110001, PR China; y Dalian No. 7 People's Hospital & Dalian Mental Health Center, No. 179 Ling Shui Lu, Gan Jing Zi District, Dalian, PR China; z The First Hospital of Hebei Medical University, No. 89 Donggang Road, Shijiazhuang 050031, PR China; aa Lanzhou University Second Hospital, Second Clinical Medical College of Lanzhou University, No. 82, Cui Ying Men, Lanzhou, Gansu 730030, PR China; ab Psychiatric Hospital of Henan Province, No. 388 Jian She Zhong Lu, Xinxiang, Henan, PR China; ac The Fourth Military Medical University affiliated Xijing Hospital, No. 17, Changle West Road, Xi'an, Shaanxi 710032, PR China; ad No. 4 People's Hospital of Liaocheng, No. 47 Hua Yuan Bei Road, Liaocheng, Shandong 252000, PR China; ae Guangzhou Brain Hospital/Guangzhou Psychiatric Hospital, No. 36 Ming Xin Lu, Fang Cun Da Dao, Li Wan District, Guangzhou 510370, PR China; af Clinical Trial Service Unit, Richard Doll Building, Old Road Campus, Roosevelt Drive, Oxford OX3 7LF, UK; ag Virginia Commonwealth University, Department of Psychiatry, Virginia Institute for Psychiatric and Behavioral Genetics, Richmond, VA 23298-0126, USA

**Keywords:** Postpartum depression, Major depressive disorder, Neuroticism, Anxiety disorder

## Abstract

**Background:**

Post partum depression (PPD) is relatively common in China but its clinical characteristics and risk factors have not been studied. We set out to investigate whether known risk factors for PPD could be found in Chinese women.

**Methods:**

A case control design was used to determine the impact of known risk factors for PPD in a cohort of 1970 Chinese women with recurrent DSM-IV major depressive disorder (MDD). In a within-case design we examined the risk factors for PPD in patients with recurrent MDD. We compared the clinical features of MDD in cases with PPD to those without MDD. Odds ratios were calculated using logistic and ordinal regression.

**Results:**

Lower occupational and educational statuses increased the risk of PPD, as did a history of pre-menstrual symptoms, stressful life events and elevated levels of the personality trait of neuroticism. Patients with PPD and MDD were more likely to experience a comorbid anxiety disorder, had a younger age of onset of MDD, have higher levels of neuroticism and dysthymia.

**Limitations:**

Results obtained in this clinical sample may not be applicable to PPD within the community. Data were obtained retrospectively and we do not know whether the correlations we observe have the same causes as those operating in other populations.

**Conclusions:**

Our results are consistent with the hypothesis that the despite cultural differences between Chinese and Western women, the phenomenology and risk factors for PPD are very similar.

## 1 Introduction

Post partum depression (PPD) is relatively common in European and US populations (Gaynes et al., 2005; Horowitz and Cousins, 2006) and, despite suggestions that Chinese cultural practices might protect women from depression in pregnancy (Pillsbury, 1978; Stern and Kruckman, 1983), epidemiological studies indicate that the prevalence of PPD is similar to elsewhere in the world (10–20%) with a one month prevalence rate of approximately 6% (He et al., 2000; Lee et al., 2001). However the clinical characteristics and risk factors for PPD have rarely been studied in China.

There are a number of known risk factors for PPD. First, a history of premenstrual symptoms is frequently found in women who develop PPD. A recent review identified five of seven studies showing this association (Payne et al., 2009). Second, a range of predisposing social factors have been identified, including low educational attainment, occupational status, stressful life events and marital difficulties (Marks et al., 1992; O'Hara et al., 1991; Paykel et al., 1980). Third, personality factors appear to be important: higher neuroticism scores increase the risk of PPD in some studies (Kumar and Robson, 1984; Marks et al., 1992) and, in one study, women scoring high on both neuroticism and introversion were about five-fold more likely to develop PPD (Verkerk et al., 2005). Finally, some have argued that PPD is associated with sensitivity to hormonal change, though the evidence for this is inconclusive (Payne et al., 2009).

We set out to investigate whether known risk factors for PPD could be found in Chinese women with MDD. Many of the risk factors also increase the risk of MDD and we were interested to determine how specific the effects would be for PPD. To do so, we used a large cohort of clinically ascertained women with recurrent MDD and compared cases with PPD to those without PPD. We asked the following questions: first, are there any clinical features that distinguish the women with PPD from those with a history of MDD who did not report PPD? Second, among women with MDD, what distinguishes those with PPD? Finally, we aimed to compare the effect of any factors we found to be significant with those reported from other studies around the world.

## 2 Methods

### 2.1 Study subjects

Data for the present study draws upon the ongoing China, Oxford and VCU Experimental Research on Genetic Epidemiology (CONVERGE) study of MDD. These analyses were based on a total of 1970 cases recruited from 53 provincial mental health centers and psychiatric departments of general medical hospitals in 41 cities in 19 provinces and four central cities: Beijing, Shanghai, Tianjin and Chongqing; 2597 controls were recruited from patients undergoing minor surgical procedures at general hospitals or from local community centers.

All cases and controls were female and had four Han Chinese grandparents. Cases and controls were excluded if they had a pre-existing history of bipolar disorder, any type of psychosis or mental retardation. Cases were aged between 30 and 60, had two or more episodes of MDD, with the first episode occurring between 14 and 50 and had not abused drug or alcohol before the first episode of MDD. Controls were chosen to match the region of origin of cases, were aged between 40 and 60, had never experienced an episode of MDD and were not blood relatives of cases. An older minimal age of controls was used to reduce the chances that they might have a subsequent first onset of MDD. The mean age (and SD) of cases and controls in the dataset was respectively 45.1 (8.8) and 47.7 (5.5).

All subjects were interviewed using a computerized assessment system, which lasted on average two hours for a case and one hour for a control. All interviewers were trained by the CONVERGE team for a minimum of one week in the use of the interview. The interview includes assessment of psychopathology, demographic and personal characteristics, and psychosocial functioning. Interviews were tape-recorded and a proportion was listened to by the trained editors who provided feedback on the quality of the interviews.

The study protocol was approved centrally by the Ethical Review Board of Oxford University and the ethics committee in participating hospitals in China.

### 2.2 Measures

The diagnoses of depressive (Dysthymia and Major Depressive Disorder) and anxiety disorders (Generalized Anxiety Disorder, Panic Disorder with or without Agoraphobia) were established with the Composite International Diagnostic Interview (CIDI) (WHO lifetime version 2.1; Chinese version), which classifies diagnoses according to the Diagnostic and Statistical Manual of Mental Disorders (DSM-IV) criteria (American Psychiatric Association, 1994). The interview was originally translated into Mandarin by a team of psychiatrists in Shanghai Mental Health Centre with the translation reviewed and modified by members of the CONVERGE team. Phobias, divided into five subtypes (animal, situational, social and blood-injury, and agoraphobia) were diagnosed using an adaptation of DSM-III criteria requiring one or more unreasonable fears, including fears of different animals, social phobia and agoraphobia that objectively interfered with the respondent's life. The section on the assessment of phobias was translated by the CONVERGE team from the interview used in the Virginia Adult Twin Study of Psychiatric and Substance Use Disorders (VATSPUD) (Kendler and Prescott, 2006).

Additional information using instruments employed from VATSPSUD, translated and reviewed for accuracy by members of the CONVERGE team, was collected on premenstrual syndrome, postnatal depression, parent child relationship, stressful life events, social life, childhood sexual abuse, smoking and neuroticism. Information on postnatal depression was assessed using an adaptation of the Edinburgh Scale (Cox et al., 1987). The history of lifetime major depression in the parents and siblings was assessed using the Family History Research Diagnostic criteria (Endicott et al., 1975). Parent–child relationship was measured with the 16-item Parental Bonding Instrument modified by Kendler (Parker et al., 2001) based on Parker's original 25-item instrument (Parker et al., 1979). Three factors were extracted from these 16 items and labeled warmth, protectiveness and authoritarianism. The dimensions of social support were assessed with a 16-item social interaction scale previously developed and used at the Institute for Social Research (Schuster et al., 1990). Measure of marital quality was an eight-item scale used in the National Comorbidity Study (Zlotnick et al., 2000). The stressful life events section, also developed for the VATSPSUD study, assessed 16 traumatic lifetime events and the age at their occurrence. The childhood sexual abuse was a shortened version of the detailed module used in the VATSPSUD study, which was in turn based on the instrument developed by Martin et al. (Zlotnick et al., 2000). Smoking was measured with the Fagerstrom Test for Nicotine Dependence (Heatherton et al., 1991), which was known to be a reliable measure of smoking behavior. Neuroticism was measured with the 23-item Eysenck Personality Questionnaire (Eysenck and Eysenck, 1975), which is an established instrument for such measurements. The Big Five Inventory was a 44-item questionnaire that was used to measure the “big five” personality traits of openness, conscientiousness, extraversion, agreeableness and neuroticism (Digman, 1990).

Premenstrual symptoms were assessed from four questions about the psychological aspects of the experience (Kendler et al., 1998). Answers, reported as “a lot”, “some”, “little” or “not at all”, were scored numerically between 4 (“a lot”) and 1 (“not at all”) and a total score obtained for each subject. Education, as an ordinal variable, is divided into 7 levels: no education or pre-school (scored 0) primary school or below (scored 1), junior middle school (scored 2), senior middle school or Technical and vocational school (in China these are equivalent) (scored 3), Adult/radio/television schooling, evening education or junior college (scored 4), bachelor degree (scored 5), master degree or above (scored 6). Occupation was classified into one of five categories: 1) executives, business owners, major and lesser professionals, 2) administrative personnel, minor professionals, clerical and sales workers, 3) skilled manual employees, 4) semi-skilled and unskilled workers, and 5) other.

Both the case and control interviews were fully computerized into a bilingual system of Mandarin and English developed in house in Oxford, and called SysQ. Skip patterns were built into SysQ. Interviews were administered by trained interviewers and entered offline in real time onto SysQ, which was installed in the laptops. Once an interview was completed, a backup file containing all the previously entered interview data could be generated with database compatible format. The backup file, together with an audio recording of the entire interview, was uploaded to a designated server currently maintained in Beijing by a service provider. All the uploaded files in the Beijing server were then transferred to an Oxford server quarterly.

### 2.3 Statistical analysis

Statistical analyses were performed using the software package SPSS 17.0 (SPSS Inc., Chicago, IL). We performed linear and logistic regression analyses to estimate the association of between postnatal depression with MDD and comorbid disorders. Coefficient values, odds ratios and 95% confidence intervals were used to quantify the strength of associations. The statistical significance for all tests was set at P < 0.05 and corrected where necessary for multiple testing using a Bonferroni correction.

## 3 Results

We obtained information about PPD for 1785 Chinese women out of a total of 1970 with recurrent MDD. 185 women (10%) had never given birth and were not included further in our analyses. The mean number of births in cases with MDD was 1.14. 548 (24%) women reported at least one occurrence of PPD (30%) with a mean number of episodes of 1.3. We identified 378 parous controls, with no history of PPD or MDD and a mean number of births of 1.21.

We began by looking for features associated with susceptibility to PPD in Western studies. We compared controls, with no history of MDD or PPD with our cases with PPD and recurrent MDD. We tested the effect of age, neuroticism, stressful life events, occupation and educational status. Table 1 gives the results of regression analyses (logisitic and ordinal) for these variables. Women with PPD were significantly younger, had higher neuroticism scores, had experienced more stressful life events, were more likely to have experienced childhood sexual abuse, had fewer years of education and had lower occupational status.

We then looked within cases with MDD to identify any features that might characterize those with PPD. We found no differences in the symptoms of MDD between those with and without PPD: none of the DSM-IV criteria differed significantly between the two groups. However we did find significant differences in rates of comorbidity and indices of severity of MDD (Table 2). With the exception of blood phobia, we found that those with PPD were more likely to experience a comorbid anxiety disorder. The effects, though, were small (odds ratios (OR) of about 1.3) and did not exceed a 5% significance threshold for panic and social phobia. A much larger effect was seen with dysthymia, where cases with PPD were about twice as likely to be affected as those without. PPD cases had a younger age of onset, had higher number of depressive episodes and a longer episode of maximal duration. However, women with PPD were actually slightly (non-significantly) less likely to meet criteria for melancholia (OR = 0.83).

We next looked at known risk factors for PPD within cases with recurrent MDD. Table 3 shows that the same risk factors we tested in our case–control comparison were significantly associated with the risk of developing PPD. Cases with PPD were younger (mean age 42 compared to 46), had lower educational levels and lower occupational status PPD. There was an effect for stressful life events (OR of 1.3), and some evidence of a dose–response effect: we found that the OR for serious personal assault gave an OR of 2.2 (P = 0.0002) and we found that childhood sexual abuse had an OR of 1.8.

## 4 Discussion

We investigated the clinical features of 1785 Chinese women with recurrent MDD and explored the effect of risk factors known to operate in Western studies. We found that, compared to controls, social factors (lower occupational and educational statuses) increase the risk of PPD, as does a history of pre-menstrual symptoms, lifetime stressful life events and higher scores on the personality trait of neuroticism. When we carried out a within MDD case study, we found that the risk factors that distinguish cases from controls also distinguish those with PPD among women with recurrent MDD. Effect sizes were almost identical in the two analyses. Our results indicated that PPD is associated with some but not all indices of severity of MDD, characterized by more episodes, a younger age of onset and longer period of maximal duration, and with more comorbid anxiety disorders.

We attempted to answer two related questions: what are the risk factors for PPD, and, among women with recurrent MDD, what distinguishes women with PPD? Our results from analyzing risk factors for PPD are consistent with reports from other parts of the world that have investigated the clinical characteristics of PPD and associated risk factors. The importance of social factors emerges as a consistent finding (Marks et al., 1992; O'Hara et al., 1991; Payne et al., 2009) (Bolton et al., 1998). We also confirmed the importance of personality (Boyce et al., 1991; Saisto et al., 2001; Verkerk et al., 2005) and stressful life events as risk factors (Beck, 1996).

Effects reported from these studies are not directly comparable to ours as they are estimated from clinical samples with PPD (rather than from a cohort of MDD patients). OR estimates from Western studies are larger than we report here. For example, women who were unemployed have twice as much chance of suffering PPD as do those who are employed (Bolton et al., 1998), compared to our estimate of 1.3. One estimate of the OR for neuroticism is 4.5, for PPD occurring 3 months after birth (Verkerk et al., 2005).

We also identified some features of MDD that distinguish those who also have PPD, namely a more neurotic depressive illness and higher rates of dysthymia. Rates of melancholia are no different between the two groups, nor does the total number of symptoms differ significantly. These observations suggest that patients with a history of PPD have a form of MDD that is more neurotic and less endogenous in nature.

The resemblance in presentation and risk factors we have observed contradicts an older literature that saw Chinese cultural practices protecting women from depression in pregnancy (Pillsbury, 1978; Stern and Kruckman, 1983). Our work indicates that PPD in China is very similar to PPD observed in the West, supporting data from epidemiological studies that report a similar prevalence rate (Lee et al., 2001; O'Hara and Swain, 1996). However our findings were obtained in patients with recurrent MDD seen in hospitals, and may not be applicable to PPD within the community. Furthermore, our data were obtained retrospectively and we do not know whether the correlations we observe have the same causes as those operating in other populations. The highly confounded nature of the risk factors we investigated will make it difficult to establish this point.

## Role of funding source

Funding for this study was provided by the Wellcome Trust; the Wellcome Trust had no further role in study design; in the collection, analysis and interpretation of data; in the writing of the report; and in the decision to submit the paper for publication.

## Conflict of interest

All authors declare they have no conflicts of interest including any financial, personal or other relationships with other people or organizations within three years of beginning the work submitted that could inappropriately influence, or be perceived to influence, their work

## Figures and Tables

**Table 1:** Risk factors for postnatal depression and recurrent MDD.

Measure	P value	OR	95% CI
Age	3.43E-40	0.89	0.88–0.91
Premenstrual symptoms (number of symptoms)	6.69E-10	1.12	1.08–1.17
Education (years)	4.15E-05	0.92	0.89–0.95
Occupation	0.025	0.94	0.89–0.99
Lifetime stressful life events (total number)	8.54E-09	1.23	1.15–1.32
Childhood sexual abuse (any)	4.18E-08	2.72	1.90–3.88
Neuroticism (per SD)	2.19E-71	1.18	1.16–1.20

The odds ratios (OR), significance (P-value) and 95% confidence intervals (95% CI) for six variables (Measure) for postnatal depression. An OR less than one indicates a lower risk of developing postnatal depression. Premenstrual symptoms are scored in arbitrary units. Neuroticism is scaled so that each unit is one standard deviation. The units for education are the number of years spent in full time education. Occupation, treated as an ordinal, is one of five categories, where executives and other professionals are scored as 5.

**Table 2:** Comorbid disorders and clinical features associated with post partum depression.

Measure	P value	OR	95% CI
Melancholia	0.12	0.83	0.66–1.04
Panic	0.06	1.40	0.98–1.99
GAD	0.01	1.37	1.08–1.76
Dysthymia	2.79E-07	2.05	1.56–2.70
Agoraphobia	0.001	1.27	1.10–1.47
Social phobia	0.09	1.13	0.98–1.30
Animal phobia	0.04	1.09	1.00–1.19
Situational phobia	0.009	1.15	1.04–1.28
Blood phobia	0.58	1.04	0.91–1.18
Age of onset	4.17E-46	0.896	0.883–0.909
Number of episodes	0.003	1.012	1.004–1.020
Maximal duration of episode	0.063	1.001	0.999–1.002

The odds ratios (OR), significance (P-value) and 95% confidence intervals (95% CI) for risk factors for postpartum depression, within cases with recurrent MDD. Results are show for nine clinical conditions and three features associated with major depression (the age of onset of MDD, the number of episodes of MDD and the duration of the longest episode of MDD).

**Table 3:** Risk factors for post partum depression within a cohort of women with recurrent MDD.

Measure	P value	OR	95% CI
Age	1.53E-13	0.95	0.03–0.06
Premenstrual symptoms	2.14E-08	1.11	1.07–1.15
Education	1.74E-04	0.88	0.83–0.93
Occupation	0.004	0.93	0.88–0.98
Lifetime stressful life events (total number)	6.40E-09	1.23	1.15–1.32
Childhood sexual abuse (any)	0.0010	1.80	1.27–2.54
Neuroticism (per SD)	7.27E-09	1.12	1.09–1.21

The odds ratios (OR), significance (P-value) and 95% confidence intervals (95% CI) for effect of six variables (listed under Measure) for postnatal depression in those with recurrent MDD. An OR of less than one indicates a lower risk of developing postnatal depression. Premenstrual symptoms are scored in arbitrary units. Neuroticism is scaled so that each unit is one standard deviation. The units for education are the number of years spent in full time education. Occupation, treated as an ordinal, is one of five categories, where executives and other professionals are scored as 5.
